# Evidence-based health technology assessments for the Brazilian National Health System (Sistema Único de Saúde, SUS) and for all*

**DOI:** 10.1590/S1516-31802009000200001

**Published:** 2009-07-06

**Authors:** Álvaro Nagib Atallah

**Affiliations:** 1 Full professor and Head of the Discipline of Emergency Medicine and Evidence-Based Medicine of Universidade Federal de São Paulo - Escola Paulista de Medicina (Unifesp-EPM). Director of Associação Paulista de Medicina (APM). E-mail: atallahmbe@uol.com.br.

Since the pioneering teachings of Brian Haynes in 1982, teaching and research on obtaining, applying and extending the best scientific evidence for decision-making in Brazil has been under development. Since 1996, with the founding of the Brazilian Cochrane Center and the creation of the Internal and Therapeutic Medicine Program of Universidade Federal de São Paulo (Unifesp), it has been possible for around 150 postgraduate students to study for master’s and doctoral degrees in this field, now called Evidence-Based Medicine. It has also been possible to make access to the scientific production of the Cochrane Collaboration available, via the Cochrane Library, to the whole population of Brazil and Latin America. This is both freely accessible and free of charge. It has been achieved with sponsorship from the Pan-American Health Organization (through the Regional Library of Medicine, Bireme) and, recently, from the Brazilian Agency for University-level Personnel Advancement (*Coordenação de Aperfeiçoamento de Pessoal de Nível Superior*, Capes), by means of initiatives and collaboration from the Brazilian Cochrane Center.

In 2004, the Brazilian Ministry of Health, through its Department of Science and Technology (DECIT), brought in the teams of the Brazilian Cochrane Center and other centers of high scientific level in this country, such as Fundação Oswaldo Cruz and Brazilian universities, to support the process of obtaining evidence through clinical research and systematic reviews, in order to provide the foundations for the Ministry’s decision-making processes. DECIT’s pioneering initiative has produced huge savings through the processes of evidence-based health technology assessments. More recently, it has innovated again, by creating the Brazilian Network for Health Technology Assessments, to bring together expertise from the whole country and offer technology and support to all areas of the national territory, at different healthcare levels.

DECIT has started to make hundreds of millions of reais available for clinical research that is formatted to meet national interests, motivated by its pioneering experience with the collaborations so far achieved and their great ethical, scientific and economic success. This research not only meets the needs of the Brazilian National Health System (*Sistema Único de Saúde*, SUS) for new knowledge, but has led to annual savings of billions of reais for the Ministry of Health and avoided economic ruin for SUS. As everyone knows, despite the known failings of SUS, it has almost unanimous and nationwide support but has few resources compared with other countries and is therefore financially very fragile. The savings have been in relation to the demands for very high prices for new technologies, mostly without any scientific basis for demonstrating their efficacy, effectiveness and safety. Such measures have created a healthcare system that is more efficient and better able to face up to sociopolitical and judicial pressures that mostly are without scientific foundation.

Today, the new epidemiology, or bluntly, epidemic of new technologies in search of market niches is very large. It can be affirmed that there are some technologies that, alone, would be capable of consuming the whole budget for SUS planned for the entire decade! And fortunately, these appear by the dozen, almost monthly. You might ask: is it fortunate or unfortunate that they appear so frequently? Well, “fortunately”, if we have the capacity to separate out those that represent real progress in relation to habitual procedures, as shown by evidence-based technology assessments, because of their advantages. The response is “unfortunately”, if we are incapable or are sufficiently irresponsible to accept them blindly and negligently. In this manner, everyone who preaches that adequate use must be made of the few public or private resources available for healthcare for the Brazilian people is bound to support an evidence-based health system, to be used as a tool for clinical, juridical and economic assessments. DECIT’s creation of the National Network for Evidence-Based Health Technology Assessments is thus a reason for the medical profession, healthcare professionals and the whole Brazilian population to feel a sense of pride.

Just to illustrate the point with a few examples, the website of the Ministry of Health’s Brazilian Bulletin for Health Technology Assessments (*Boletim Brasileiro de Avaliação de Tecnologias em Saúde*, BRATS) shows that one systematic review on a product of debatable clinical usefulness gave rise to a reduction in the annual budget requirement of around 800 million reais four years ago. In other words, the review has already prevented a budgetary impact of around three billion reais. In another example, one of the monoclonal antibodies recommended for psoriasis, if not judiciously used, could imply costs of five billion reais per week to treat the one million patients in this country with this disease.

Within this general context, the judicialization of medicine is already costing the public purse an estimated one billion reais per year. This occurs when the courts of law determine that SUS and state health departments should release the use of treatments. The judges who authorize prescriptions for such treatments with the aim of “resolving problems” ignore the evidence relating to their effectiveness and safety.

It is therefore more than necessary for health scientists and professionals within the Brazilian judiciary to join forces in a wide-ranging dialogue to provide real benefit for healthcare and for the preservation of a health system that is unique and needs to be improved but, above all, safeguarded! With this aim, the First Brazilian Congress of Evidence-Based Medicine and the Right to Health has been successfully held in Brasília, organized and sponsored by judicial and healthcare entities. This has enabled great sharing of ideas, ideals and perspectives for a better country. The lectures can be seen at the site: http://centrocochranedobrasil.org.br

